# Regular Consumption of Cocoa and Red Berries as a Strategy to Improve Cardiovascular Biomarkers via Modulation of Microbiota Metabolism in Healthy Aging Adults

**DOI:** 10.3390/nu15102299

**Published:** 2023-05-13

**Authors:** Joaquín García-Cordero, Alba Martinez, Carlos Blanco-Valverde, Alicia Pino, Verónica Puertas-Martín, Ricardo San Román, Sonia de Pascual-Teresa

**Affiliations:** 1 Departamento de Metabolismo y Nutrición, Instituto de Ciencia y Tecnología de Alimentos y Nutrición (ICTAN-CSIC), C/José Antonio Novais, 10, 28040 Madrid, Spain; j.garcia@ictan.csic.es (J.G.-C.);; 2 Hospital 12 de Octubre, 28041 Madrid, Spain; 3 Facultad de Educación, Universidad Internacional de la Rioja, 26006 Logroño, Spain

**Keywords:** flavanols, anthocyanins, cardiovascular, TMAO, short-chain fatty acids, bile acids

## Abstract

The aim of the present study was to analyze the effects of cocoa flavanols and red berry anthocyanins on cardiovascular biomarkers, such as homocysteine, angiotensin-converting enzyme (ACE), nitric oxide (NO), flow-mediated vasodilation (FMD), blood pressure and lipid profile. Additionally, we aimed to ascertain their possible interactions with microbiota related metabolites, such as secondary bile acids (SBA), short-chain fatty acids (SCFA) and trimethylamine N-oxide (TMAO). A randomized, parallel-group study, single-blind for the research team, was performed on 60 healthy volunteers between the ages of 45 and 85, who consumed 2.5 g/day of cocoa powder (9.59 mg/day of total flavanols), 5 g/day of a red berry mixture (13.9 mg/day of total anthocyanins) or 7.5 g/day of a combination of both for 12 weeks. The group that had consumed cocoa showed a significant reduction in TMAO (*p* = 0.03) and uric acid (*p* = 0.01) levels in serum, accompanied by an increase in FMD values (*p* = 0.03) and total polyphenols. corrected by creatinine (*p* = 0.03) after the intervention. These latter values negatively correlated with the TMAO concentration (R = −0.57, *p* = 0.02). Additionally, we observed an increase in carbohydrate fermentation in the groups that had consumed cocoa (*p* = 0.04) and red berries (*p* = 0.04) between the beginning and the end of the intervention. This increase in carbohydrate fermentation was correlated with lower levels of TC/HDL ratio (*p* = 0.01), systolic (*p* = 0.01) and diastolic blood pressure (*p* = 0.01). In conclusion, our study showed a positive modulation of microbiota metabolism after a regular intake of cocoa flavanols and red berry anthocyanins that led to an improvement in cardiovascular function, especially in the group that consumed cocoa.

## 1. Introduction

The last decades have seen a demographic shift in age groups, with a marked increase in the population’s average age [1,2]. The cardiovascular system is one of the most commonly affected by this process, as ageing is associated with an increased risk of developing hypertension, atherosclerosis and myocardial infarction [3]. After cancer, cardiovascular diseases (CVD) represent the second leading cause of death worldwide, with the majority of these being reported in people over the age of 60 [4]. During ageing, dysfunction of the vascular endothelium occurs, along with increased arterial thickening and stiffness [5]. On the other hand, age brings about a reduction in endothelial nitric oxide synthase (eNOS) activity responsible for synthesizing nitric oxide (NO), a critical vasodilator that regulates vascular tone and inhibits vascular inflammation [3,5]. This increase in both mortality and morbidity caused by CVD has made it necessary to search for new strategies to reduce the prevalence of these diseases and lessen the harmful effects of ageing. Dietary polyphenols have been shown to be effective in combatting the effects of ageing, especially in preventing the decline of cognitive function [6,7] and the development of CVD [8,9]. Polyphenols are a large group of secondary metabolites in plants exhibiting a multitude of beneficial attributes for the organism. However, the most important factor to consider for the present work is their anti-aging properties on the vascular system and their regulatory effect on major inflammatory and ROS-dependent signaling pathways associated with senescence [9].

Flavanols, a type of polyphenol belonging to the group of flavonoids, have been shown to be particularly promising compounds in preventing cardiovascular age-related dysfunctions [10]. Epidemiological studies on flavanol-rich foods, such as tea [11], apples [12], red wine [13] and chocolate [14], have observed positive trends in the association between a high consumption of these foods and reduced CVD risk. This association has also been shown in people who had previously suffered a cardiovascular event [15]. In fact, a meta-analysis conducted by Lin et al. (2016) found that cocoa flavanol intake in adults significantly improved various cardiovascular biomarkers, such as fasting insulin, insulin resistance, triglycerides (TG), HDL-C, c-reactive protein (CRP) and VCAM-1 [16]. In a similar way, the meta-analysis of Arab et al. (2009) showed that tea consumption (three cups versus lees than one cup per day) produced a reduction in the risk of suffering a hard attack by 21% [17]. In the light of this evidence, the European Food Safety Authority (EFSA) stated, in a 2012 health claim, that 200 mg cocoa flavanols per day might help in maintaining a normal blood flow and endothelium-dependent vasodilation [18].

Other flavonoids that have shown beneficial effects on cardiovascular health are anthocyanins, red to blue compounds that are present in various fruits and vegetables, including red fruits [19]. Several clinical studies have found a positive association between anthocyanin intake and cardiovascular biomarkers. For example, a randomized, double-blind trial carried out on 150 subjects aged 40–65 with hypercholesterolemia, showed that the consumption of a purified anthocyanin mixture (320 mg/day) significantly reduced serum levels of VCAM-1, LDL-C, CRP and plasma IL-1β, while increasing serum levels of HDL-C, compared with the placebo [20]. Another randomized trial on 146 hypercholesterolemic subjects aged 40–65 also found a reduction in platelet chemokines in plasma after the consumption of 320 mg of purified anthocyanins, compared with the placebo. [21]. In previous work from our group, it was shown that anthocyanins and their metabolites can inhibit monocyte chemoattractant protein 1 (MCP-1) secretion [22,23] and endothelial adhesion molecules, VCAM-1 and ICAM-1 [22]. Moreover, a meta-analysis conducted by Angelo et al. (2018) found that the intake of berries reduced total cholesterol (TC), LDL-C, TG and blood pressure, while increasing the level of HDL-C, suggesting a beneficial effect on the control of CVD risk factors [24].

Additionally, polyphenols could influence cardiovascular health positively due to their effect on gut microbiota. Changes in microbiota composition and metabolism are associated with numerous diseases, including CVD [25]. For instance, trimethylamine N-oxide (TMAO) is a gut microbiota-dependent metabolite formed following ingestion of choline-rich foods and whose circulating levels have been associated with CVD risk in large scale clinical studies [26]. Another pathway in which microbiota could modulate a host metabolism is through the production of secondary bile acids (SBA) and short-chain fatty acids (SCFA). Many of these SBAs and SCFAs act as hormone-like molecules following gut absorption, interacting with a variety of host receptors involved in inflammation,# and lipid and glucose metabolism [25]. Perturbations in the dynamic between diet, gut microbiota and SBA/SCFA may increase CVD risk. Haeusler et al. (2013) showed that increased levels of 12α-hydroxylated bile acids, cholic acid, deoxycholic acid and their conjugated forms in plasma were associated with higher insulin, proinsulin, glucose, glucagon and TG and lower HDL-C levels in plasma [27]. On the other hand, SFCAs lowered blood pressure in mice via endothelial G protein-coupled receptor 41 [28]. Consumption of both flavanols [29] and anthocyanins [30] has been shown to regulate gut microbiota composition in a positive way; for instance, the ingestion of cocoa has been associated with an increase in Lactobacillus and Bifidobacterium abundance and a reduction in species from the Clostridium genus [31]. These changes are associated with a decrease in concentrations of CRP and inflammation, thus lowering the cardiovascular risk [29].

Given the increase in CVD in the elderly population and the need to find new ways to prevent this, the aim of this research was to study the activity of red fruit anthocyanins and cocoa flavanols on cardiovascular biomarkers, including FMD, blood pressure, lipid and glycemic profile and angiotensin-converting enzyme (ACE). Furthermore, our aim was to examine whether anthocyanins and flavanols alone or in combination correlated with an improvement in these CVD biomarkers and with the gut microbiota metabolism measured as TMAO, SCFA and BA serum levels.

## 2. Materials and Methods

### 2.1. Study Design and Ethical Considerations

This was a randomized, single-blind, parallel-group study lasting 12 weeks. The volunteers were assigned to three groups (referred to as diets throughout the study) according to the consumed product: group 1 consumed 5 g/day of a mixture of red berries (RB); group 2 consumed 2.5 g/day of a polyphenol-rich cocoa powder (C) and group 3 consumed 7.5 g/day of a mixture of cocoa and red berries (RB+C). The product was kindly prepared and supplied by Salengei^®^ (Barcelona, Spain).

**Figure 1:**
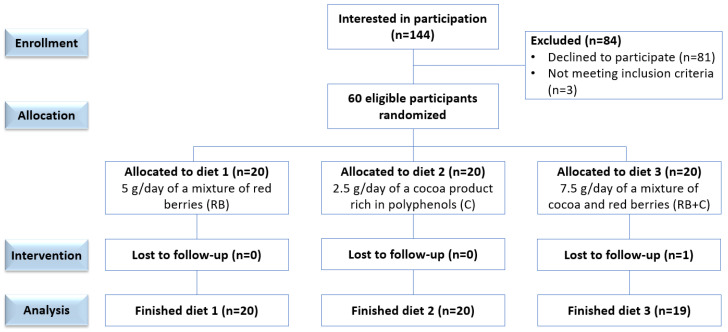
Flow diagram of the randomized, single-blinded, parallel trial.

Of the 144 volunteers that were approached, only 60 were included in the study after giving their written consent. They were randomly assigned to one of the three diets, as presented in the flow diagram of the intervention study (Figure 1). Only one subject withdrew from the study for personal or health reasons, and 59 completed it successfully. Volunteers came to the Human Nutrition Unit at ICTAN-CSIC on three different days for sample and data collection. The first visit corresponded with the personal interview to check eligibility. The second visit was considered the baseline or start of the study, and the third visit was the end of the 12-week intervention. On each visit, a 20 mL blood sample, early morning urine, weight, height and waist circumference (anthropometric measurements), blood pressure and 24-h diet records were collected. At the beginning of the intervention, and after revising the rules of the study to all volunteers, they were provided with the product to be consumed. In every case, volunteers were contacted by telephone to check compliance every two weeks, in order to resolve any queries and encourage completion of the study. From the three-day dietary collections (which included two mid-weekdays and one weekend day), we also recorded the frequency of consumption of the main sources of anthocyanins, flavanols, caffeine and theobromine in order to calculate the total daily intake. The purpose of this was to check that the daily intake of cocoa powder represented approximately 75 mg of theobromine and 3 mg of caffeine. We considered it important to monitor the dietary consumption of methylxanthines, theobromine and caffeine, for two reasons. One is that some studies have associated high blood pressure with caffeine consumption in certain individuals with a genetic predisposition or a lower-metabolizing rate for caffeine. The second is that there is a scientific controversy as to weather theobromine or flavanols in cocoa are responsible its cardiovascular protective effect.

All personal data (biological samples and neuropsychological tests) were processed in accordance with the Organic Law 3/2018, of 6 December, on the Protection of Personal Data (https://www.boe.es/eli/es/lo/2018/12/05/3 (accessed on 5 July 2019)), using codes to ensure confidentiality and guarantee anonymity from the moment of recruitment. The study was approved by the Bioethics Committee of the Spanish National Research Council (CSIC), the Ethics Committee for Clinical Research of the Hospital Universitario Puerta de Hierro-Majadahonda (Madrid, Spain) (Acta no 01.07) and the Hospital Universitario 12 de Octubre (Madrid, Spain) (Acta no17/117). All subjects gave their written, informed consent after receiving oral and written information about the study. ClinicalTrials.gov trial registration—NCT04348162. The clinical trial was conducted in accordance with the principles of good clinical practice (Royal Decree 1090/2015 of 4 December) and the Declaration of Helsinki (http://www.wma.net/es/30publications/10policies/b3/index.html (accessed on 17 August 2017)).

### 2.2. Subjects

Volunteer were recruited by the research team in the area of Madrid, through posters and online dissemination through Universidad Complutense de Madrid (UCM), CSIC channels and social networks. We selected aging adults 45 to 85 years old, either men or postmenopausal women (considering one year with amenorrhea) as potential candidates. Participation in the study was voluntary. Exclusion criteria were body mass index (BMI) lower than 20 or higher than 30, more than five cigarettes per day smoked, familial hypercholesterolemia (serum triglycerides > 250 mg/dL or cardiovascular risk index total cholesterol/HDL-C > 6), chronic diseases (diabetes, liver, kidney, etc.), anticoagulants, unavailability to resume intake of supplements containing polyphenols, phytosterols, vitamins or minerals during the study, or antibiotic prescription in the last three months. Product aversion and a score below 28 on the Folstein Mini-mental state examination (MMSE), above 6 on the Functional Activities Questionnaire (FAQ) and below 10 on the Beck depression scale were also considered as exclusion criteria. Once included in the study, the volunteers were randomized to consume one of the three diets using the Microsoft^®^ Excel 2016 program and using as key criteria age, BMI and gender, before the first visit. In every case, participants in the study were asked to maintain the same lifestyle and diet as before the intervention.

### 2.3. Characterization of Cocoa and Red Berry Powders

The characterization of the dietary products has been described in a previous publication of our group [32,33]. In short, the semi-defatted cocoa powder, kindly supplied by Salengei^®^ (Barcelona, Spain), as were the rest of the products, was from organic farming and of high polyphenol content. It was sugar-, sweetener- and emulsifier-free and contained 7899 mg of total polyphenols, 668 mg of theobromine and 275 mg of caffeine per 100 g cocoa powder. The cocoa powder consumed by the volunteer provided a daily intake of 197.5 mg total polyphenols. Additionally, the cocoa powder contained 383.6 mg of total flavanols per 100 g, which provided a daily intake of 9.6 mg flavanols, including 4.7 mg epicatechin. The second product was a red berry mixture made of a combination of pure dried redcurrants (33.3%), blackcurrants (33.3%), raspberries (16.7%) and blueberries (16.7%). The determination of total polyphenols and anthocyanins showed that the red berry mixture contained 2079 mg of total polyphenols and 277.7 mg of total anthocyanins in 100 g of the product, which provided a daily intake of 104 mg total polyphenols and 13.9 mg total anthocyanins, including 4.8 mg delphinidin-3-O-rutinoside and 3.5 mg cyanidin-3-O-rutinoside. There was no trace of other substances that could adulterate the final product. Further information about energy value and nutrient composition is provided in the supplementary material (Tables S1 and S2). In both products, the method of use was to dissolve one tablespoon per day in water, vegetable milk, juice, yoghurt or cereals with the main meal. The tablespoon was provided for each product and the size and the best way to obtain the exact dose was explained to each volunteer before the start of the intervention.

### 2.4. Determination of the ACE Activity

The analysis was performed using a modified Friedland and Silverstein fluorometric assay [34]. Starting from a commercial stock of 0.1 U of porcine ACE powder, serial dilutions of between 5 U/L and 50 U/L were made for the standard curve in the phosphate buffered saline (PBS) solution with the pH adjusted to 8.3. We used the tripeptide Hippuryl-Histidine-Leucine as a substrate for the enzyme, dissolving it in PBS using a sonicator to obtain a final concentration of 2.3 mM. We added 120 μL of the tripeptide solution to 30 μL of each diluted sample to start the reaction, incubating it for 20 min at 37 °C. Next, 150 μL of 3 M NaOH was added to stop the reaction. Then, 100 μL of 0.2% orthophthalaldehyde in methanol was added and incubated in the dark for 10 min at 37 °C. Finally, we added 150 μL of 3 M HCl to each sample and centrifuged at 2000× *g* for 2 min at 4 °C. The samples were analyzed in a Biotek Synergy HT Microplate Reader, employing an excitation wavelength of 360 nm and a readout of 500 nm.

### 2.5. Quantification of Nitric Oxide in Plasma

The quantification of nitric oxide in plasma samples was made following the method of Giustarini et al. (2004) [35] with modifications. A dilution of sodium nitrite in ultrapure water was used for the standard curve, with concentrations between 1.5 μM and 75 μM. First, 5 μL of N-ethylmaleimide was added to 100 μL of plasma or the control, followed by the addition of 400 μL of 62.5% ethanol solution. After shaking, we introduced 500 μL of modified Griess reagent to each of the samples and incubated the solution in the dark for 30 min. To deproteinize the mixture, 200 μL of cold TCA was added to a final concentration of 3% m/v. Next, the sample was centrifuged at 10,000× *g* for two minutes, and the supernatant was collected in a 96-well flat-bottom plate (200 μL per well, in triplicate). The color change was quantified on the plate of a Biotek Synergy HT Microplate Reader, reading at 540 nm.

### 2.6. Measurement of Flow-Mediated Dilatation (FMD)

FMD measurement was performed with a Toshiba Aplio 500 platinum ultrasound machine in the left humeral artery, between 5 and 10 cm above the elbow. The volunteer was placed in a supine position, and had to fast and abstain from smoking for at least eight hours. To induce ischemia, a cuff was placed distal to the measurement site for five minutes, exerting a pressure at least 50 mmHg higher than the systolic pressure. Measurements were taken of baseline arterial diameter and peak arterial diameter after reactive hyperemia. The diameters were measured from the interface between the blood and the beginning of the intima-media boundary of the vascular wall at the end of diastole. FMV was calculated as the percentage change between the two diameters [36]. This procedure was performed by professionally trained health personnel at Hospital 12 de Octubre Madrid.

### 2.7. Determination of Trimethylamine Oxide Levels in Serum

The analysis of trimethylamine oxide levels was carried out using a modified method by Li et al. (2009) [37] adapted to human serum samples. First, we added 0.4 mL of 7.5% trichloroacetic acid (TCA) to 0.5 mL serum samples and put it in centrifugation for 15 min at 6000 rpm and a temperature of 4 °C. After the first centrifugation, we collected the supernatant and added 0.3 mL of 5% TCA. Then, we applied a second centrifugation for 15 min under the same conditions and collected the supernatant. This process was repeated a third time, leaving the samples in centrifugation for 15 min but in this case at 13,000 rpm and a temperature of 4 °C. The supernatant of the three extractions was collected and pooled to be analyzed by ion exchange chromatography with a conductivity detector, a mobile phase of 4 mM meta-sulfonic acid, a metrosep C6 column and a flow rate of 1 mL/min. A standard curve with trimethylamine oxide as the external standard was previously analyzed at a concentration range between 6.4 to 51.2 µM.

### 2.8. Determination of Plasmatic Levels of Short-Chain Fatty Acids and Bile Acids

The extraction of the SCFAs was performed using a modified method by Folch et al. (1957) [38]. First, we added 300 μL of 0.5% phosphoric acid to 400 μL of plasma. Next, we added 100 μL of 4-methyl valeric acid as the internal standard and centrifuged for 15 min at 12,000 rpm and a temperature of 4 °C to facilitate protein precipitation. After centrifugation, we extracted 750 μL of supernatant which was mixed with 200 μL butanol and vortexed for 30 s (×3). The supernatants were then placed in a vial with an insert for gas chromatography with a flame ion detector (GC-FID) analysis according to the protocol proposed by Zhao et al. (2007) [39]. The separation was carried out using a 100% polyethylene glycol column, helium as carrier gas and a constant flow rate of 1.5 mL/min associated. In addition to calculating the different SCFAs and the sum of all of them, we calculated the fermentation indexes. These indices were: fermentation index A (FIA), which refers to the fermentation of carbohydrates and is calculated as the difference of acetic acid minus propionic and butyric acid divided by total SCFA value, and fermentation index B (FIB), which refers to the fermentation of proteins and is obtained from the sum of the concentration of iso-butyric and isovaleric acid [40].

For the extraction of the BA in human plasma samples, we conducted a method set up by our group. We started by adding 300 μL of H_2_O: ACN 1:1 and 15 μL of CDCA-d4 as the internal standard (10 μg/mL of CDCA-d4 in H_2_O: MeOH 1:1) to 200 μL of plasma, then these samples were vortexed at 1300 rpm for 30 s and left at room temperature for 10 min. After this time, the samples were shaken again in the vortex and centrifuged for 5 min at 1300 rpm and a temperature of 4 °C (×2). Next, the supernatant was collected and evaporated under nitrogen atmosphere. The dry extract was reconstituted with 100 μL of H_2_O: MeOH 1:1, vortexed and centrifuged for five minutes at 1000 rpm and 4 °C. This last supernatant was analyzed and BA quantified by HPLC-QQQ-MS, employing as the standard curve a commercial standard of human BA which included Lithocholic acid (LCA), Chenodeoxycholic acid (CDCA), Deoxycholic acid (DCA), Glyco-deoxycholic acid (GDCA), Glycolic acid (GCA), Tauro-deoxycholic acid (TDCA), Taurocholic acid (TCA), Hyo-deoxycholic acid (HDCA), Urso-deoxycholic acid (UDCA), Cholic acid (CA) and Hyo-cholic acid (HC) at concentrations between 5 to 0.001 µg/mL. The separation was carried out using a Kinetex XB-C18 100A (Phenomenex Spain, Madrid) as column and a Phenomenex UHPLC C18 as precolumn, ammonium acetate 2 mM in H_2_O and ACN: MeOH (1:1) as mobile phases and a constant flow rate of 1 mL/min at a temperature of 50 °C.

### 2.9. Determination of Other Biochemical Parameters

Total serum protein was determined by the Bradford method using bovine serum albumin (BSA) as the external standard. The serum samples were diluted 200-fold before analysis. The absorbance was measured at 595 nm on a Biotek Synergy HT Microplate Reader (Agilent, Santa Clara, CA, USA). The determination of total polyphenols and creatinine in urine has been described in a previous publication of our group [7], performing a standard curve with commercial gallic acid from 300 to 4.7 µg/mL in ultrapure water. To normalize the data obtained for the polyphenols, we determined the concentrations of urine creatinine by a colorimetric reaction. Urine samples were diluted 20-fold and 10 µL pipetted into a 96-well, flat-bottom plate. Next, 200 µL 0.1% picric acid and 15 µL of NaOH were added. After a 15-min incubation, the plate was read at an absorbance of 500 nm by using a Biotek Synergy HT Microplate Reader.

### 2.10. Statistical Analysis

Statistical analysis was performed with IBM SPSS version 28 software (SPSS, Inc., Chicago, IL, USA, 2020). Data were expressed as mean ± standard error (M ± SE). The level of significance was set at *p* < 0.05. Boxplot analysis was carried out to detect outliers and to determine the dispersion and symmetry of the data. The normality of distribution and homogeneity of variance were evaluated using the Kolmogorov-Smirnov and Levene tests, respectively. A Kruskal-Wallis test was applied when comparing means of the three diets at baseline and at the end of the 12-week intervention, and a Wilcoxon test to compare the results between baseline and after intervention 12 weeks in each diet. Bivariate correlations were performed, segmented by sex, to test the association between the concentrations of BA, SCFR and total polyphenols with the different metabolic and cardiovascular biomarkers. Scatter plots were constructed to graphically represent the relationship between paired variables. Additionally, the Benjamini-Hochberg procedure was used to correct for the false discovery rate (FDR). Due to the large number of correlation models run for this analysis, we selected an FDR of 10%.

## 3. Results

### 3.1. Baseline Population Characteristics

The characteristics of our population have been defined in our previous study [7]. Further information about population characteristics is provided in the supplementary material (Table S3). In short, no significant differences between groups at baseline was found in age, sex, height, or percentage of smokers. Only 20% of our study population were considered to be chronic smokers.

### 3.2. Cardiovascular Parameters

**Figure 2:**
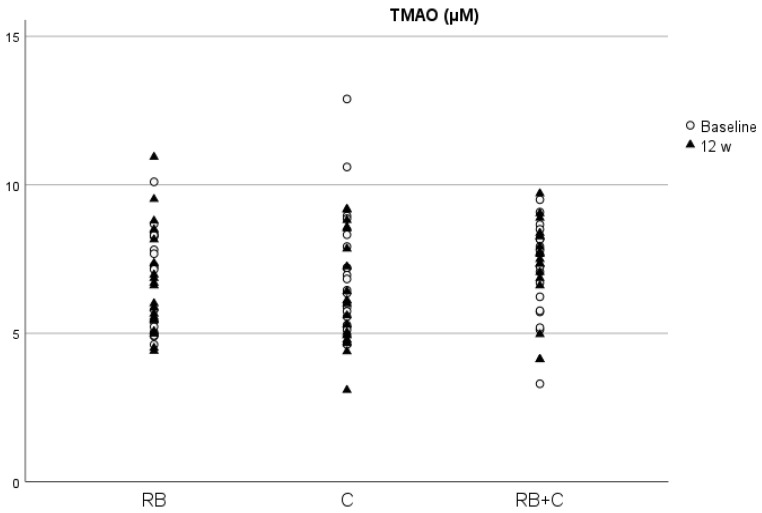
Concentrations of TMAO in serum at baseline and at the end of the intervention in the three diets. (RB) Red Berries, (C) Cacao, (RB+C) Mixture.

**Figure 3:**
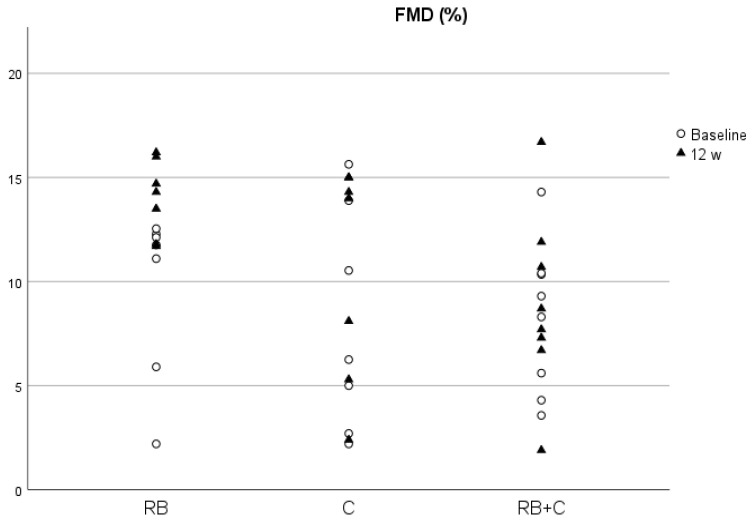
FMD levels at baseline and at the end of the intervention in the three diets. (RB) Red Berries, (C) Cacao, (RB+C) Mixture.

The *p*-values obtained from the comparison between diets at the baseline is provided in the supplementary material (Table S5). We found no significant difference between the groups at baseline in each of the parameters analyzed. Additionally, we found no significant differences between groups at the end of the intervention in the concentrations of total serum protein, NO, homocysteine, TMAO and ACE activity corrected by total protein (Table 1). Between visits, we only observed a statistically significant increase for FMD values, in the cocoa powder group (*p* = 0.03) and a decrease in TMAO levels after the 12-week intervention in the C group (*p* = 0.03) (Figure 2 and Figure 3). We did not find any other significant change in any of the analyzed parameters between baseline and 12-weeks for any of the diets.

On the other hand, FMD values were significantly different between the C and RB groups at the end of the intervention, with higher values observed in the C group. The results from the blood pressure and heart rate analysis are provided in the supplementary material (Table S4). Significant increases in heart rate values were observed in the RB (*p* > 0.00) and C (*p* = 0.01) group after the intervention.

### 3.3. Metabolic Parameters

As with the cardiovascular parameters, we did not find any significant differences between groups at the start of the study in each of the parameters analyzed (Table S5). Table 2 shows the results from the analysis of lipid metabolism biomarkers, glucose, iron and CPR levels in serum at baseline and at the end of the intervention. There were no significant differences between any of the groups at the endpoint and between visits for any of the parameters.

As it can be seen in Table 3, there was a significant reduction in the concentration of creatinine (*p* = 0.03) and uric acids (*p* = 0.01) levels in the C group after the 12-week intervention.

### 3.4. Polyphenol Levels Corrected by Creatinine

Table 4 depicts the statistical results of the creatinine-corrected polyphenol levels. There were significant differences between the C and RB+C groups (*p* = 0.04) at the end of the 12-week intervention, with the group that had consumed the cocoa product exhibiting the largest increase in polyphenol levels in comparison with the groups that had consumed the mixture of red berries and cocoa. When examining the differences within the groups, we observed a significant increase in polyphenol plasma levels in the C group (*p* = 0.03), while the in the case of the RB group the increase in total polyphenols remained close to significance (*p* = 0.06).

### 3.5. Analysis of Bile Acids

Table 5 shows the mean and standard deviation for the 10 principal BAs found in the serum samples: CDCA, DCA, GDCA, GCA, TDCA, TCA, Glycokeno-deoxycholic acid (GCDCA), Glycourso-deoxycholic acid (GUDCA), Tauroquene-deoxycholic acid (TCDCA) and Glyco-lithocholic acid (GLCA), as well as the sum of the primary bile acids, secondary bile acids and bile acid profile totals for each of the volunteers at baseline and at the end of the 12-week intervention. No significant differences were observed at baseline between the groups (Table S5), so we can confirm that all groups commenced the study with similar levels. After the 12-week intervention, we found significant differences only in the case of CDCA between the RB and C groups, with the group that had consumed the red berry mixture exhibiting the largest increase in CDCA concentrations in blood. As for the significant differences within the groups, we only found statistically significant differences in DCA in the RB group, where concentrations of this BA increased after the intervention with the red berry mixture, and a nearly significant difference in CDCA also in the RB group, with an appreciable increase after the intervention.

### 3.6. Analysis of Short-Chain Fatty Acids

Table 6 shows the means and standard deviations of the SCFAs analyzed by GC-FID normalized to the serum protein levels obtained in the same sample: acetic (ACE), propionic (PRO), iso-butyric (ISOB), butyric (BUT), isovaleric (ISOV), valeric (VAL), caproic (CAP) and heptanoic acids (HEP), as well as the sum of all of them and the FIA and FIB. As with the BA analysis, we did not find any significant differences at baseline between the groups (Table S5), so we assumed that the three groups started the study with similar levels.

**Figure 4:**
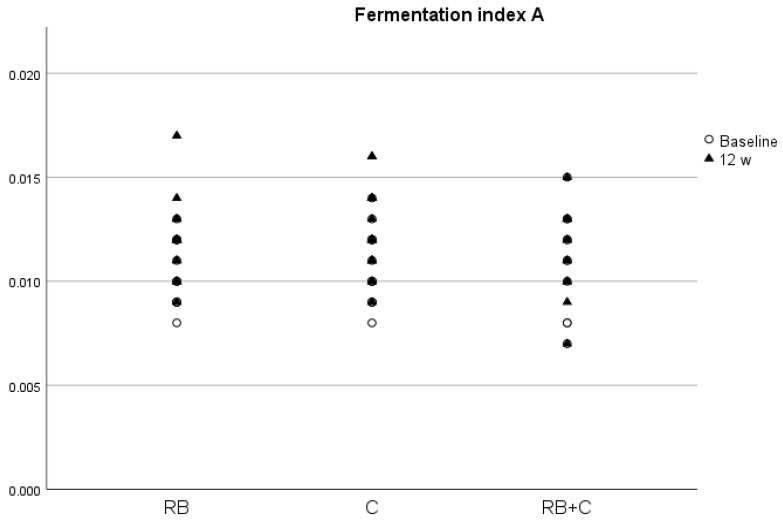
Fermentation index A (FIA) at baseline and at the end of the intervention in the three diets. (RB) Red Berries, (C) Cacao, (RB+C) Mixture.

We only observed statistically significant changes between groups at the end of the intervention for ISOV, with a significantly higher concentration in group C compared with group RB+C, and for CAP, with a significantly higher concentration in group RB compared with group RB+C. There was a slight increase in all SCFAs after the intervention with the food products, except in the case of BUT in the C and RB+C groups. However, there was only a significant increase in FIA ratio in the RB and C groups (*p* = 0.04 and *p* = 0.04, respectively) (Figure 4), with a nearly significant increase in ACE and total SCFAs in the group that had consumed red berry anthocyanins (*p* = 0.06 and *p* = 0.06, respectively).

### 3.7. Correlations between Polyphenol Levels, Bile Acids, Short-Chain Fatty Acids and Cardiovascular Biomarkers

Table 7, Table 8 and Table 9 show the main results obtained after analyzing the correlations between the different biochemical parameters related to cardiovascular health, SCFA and BA concentrations with polyphenol levels corrected by creatinine at the end of the intervention and divided by sex. Only a negative correlation between polyphenol levels and TMAO concentrations (*p* = 0.02) was found, resulting in high polyphenol levels being associated with lower TMAO levels.

Additionally, we analyzed the correlations between concentrations of SCFA and biochemical parameters of cardiovascular health. In men, we found positive correlations between TMAO and HEP concentrations (R = 0.61, *p* = 0.04), and between FMD values and CAP (R = 0.72, *p* = 0.045) concentrations, with a negative correlation found between NO and CAP (R = −0.54, *p* = 0.046). In women, we found positive correlations between ACE/total protein and VAL concentrations (R = 0.47, *p* = 0.01); ISOV and TC (R = −0.42, *p* = 0.03) and HDL (R = −0.49, *p* = 0.00); iron concentrations and FIA (R = 0.46, *p* = 0.01); and between FMD values and ISOV concentrations (R = 0.77, *p* = 0.02), with negative correlations found between FIA and TC/HDL (R = −0.49, *p* = 0.01), SBP (R = −0.45, *p* = 0.01) and DBP (R = −0.48, *p* = 0.01), and between FIB and SBP (R = −0.39, *p* = 0.04) and DBP (R = −0.39, *p* = 0.04).

The correlations between BA and biochemical parameters of cardiovascular health were also analyzed. In this case, we found positive correlations in men between glucose concentrations and CDCA (R = 0.61, *p* = 0.04), DCA (R = 0.36, *p* = 0.045) and GDCA (R = 0.69, *p* = 0.03); between creatinine concentrations and DCA (R = 0.57, *p* = 0.03); TG and GDCA (R = 0.61, *p* = 0.03), TCA (R = 0.76, *p* = 0.01), TCDCA (R = 0.68, *p* = 0.02) and GLCA (R = 0.6, *p* = 0.04); SBP and total BA (R = 0.76, *p* = 0.03) and secondary BA (R = 0.69, *p* = 0.04); DBP and secondary BA (R = 0.741, *p* = 0.022); and between HR and secondary BA (R = 0.73, *p* = 0.03); with a negative correlation between iron concentrations in serum and TCDCA (R = −0.57, *p* = 0.04). In women, we found positive correlations between TC and TCDCA (R = 0.43, *p* = 0.02), with negative correlations between DBP and GDCA (R = −0.43, *p* = 0.01), primary (R = −0.45, *p* = 0.04) and secondary BA (R = −0.56, *p* = 0.04). These findings were consistently significant after Benjamini-Hochberg correction. 

## 4. Discussion

Scientific evidence suggests that both flavanols [11,12,13,14,15,16,17,18] and anthocyanins [19,20,21,22,23,24] have cardioprotective properties. Flavonoids in general present important antioxidant and chelating properties, inactivating ROS and preventing the oxidation of LDL and reducing inflammation of the blood vessel wall by the inhibition of the influx of leucocytes. Flavonoids also decrease the activity of enzymes related to increased ROS production and oxidative damage, such as xanthine oxidase and NADPH oxidase, and inflammation, such as 15-lipoxygenase and COX-2, leading to a reduction in the synthesis of pro-inflammatory molecules [41]. Beyond their antioxidant and anti-inflammatory effects, flavonoids also protect the cardiovascular system by regulating other metabolic pathways. Flavonoids have been shown to suppress the activity of the 3-hydroxy-3-methylglutaryl-coen-zyme A reductase (HMG-CoA), an enzyme that plays a key role in the synthesis of cholesterol and whose inhibition leads to lower intracellular cholesterol and increased expression of LDL receptors. Moreover, flavonoids exhibit some anti-obesity properties, improving the lipid profile and decreasing insulin resistance [16,20]. Regarding vascular integrity, some flavonoids have been shown to help seal and reinforce blood vessel walls by enhancing collagen synthesis [41]. Bearing in mind the predicted increase in the elderly population in the coming decades and the high prevalence of cardiovascular disease in this population, it is urgent to find new strategies to combat these pathologies. Therefore, this work was aimed at assessing the positive and anti-ageing effects on cardiovascular health of chronic consumption of cocoa flavanols, red berry anthocyanins or a combination of both for 12 weeks. No statistically significant differences were shown between groups regarding BMI, gender, age, height and percentage of smokers, in our study population, confirming that the randomization was carried out correctly. Furthermore, we did not observe any significant differences at the start of the intervention between the three parallel groups in any of the different cardiovascular and metabolic biomarkers, total polyphenol, and BA and SCFA concentrations.

In vitro and in vivo studies using both flavanols [42,43,44] and anthocyanins [45,46] have shown that these phenolic compounds could inhibit ACE activity by competing with the substrate for the active site. Some clinical trials have also shown an ACE inhibitory activity after the consumption of flavanols or anthocyanins [45,47]. With these results in mind, we expected that the ingestion of a combination of cocoa flavanols and red berry anthocyanins could enhance this inhibitory effect, and thus be a more effective treatment against hypertension or other cardiovascular diseases. However, at the end of the 12-week intervention, we did not find an improvement in ACE activity corrected by total protein with any of the different food products and with only a slight decrease in the C group. These results could be due to the low dose of flavanols (9.59 mg/day) and anthocyanins (13.9 mg/day) given to the volunteers. The dose might have been insufficient to reach the concentrations necessary to produce a significant effect on ACE activity, as the in vitro study conducted by Actis-Goretta et al. on rat kidney tissue stated that the inhibition of ACE activity was dependent on the flavanol content of chocolate, as high-procyanidin chocolate (2.22 mM of flavanols) statistically reduced the ACE activity more significantly than low-procyanidin chocolate (1.1 mM of flavanols) [42]. For example, in an acute clinical trial conducted by Persson et al. (2011) on 16 healthy subjects, a significant inhibition of ACE activity was observed three hours after the intake of 75 g of dark chocolate (72% cocoa) [44]. In the case of anthocyanins, a double-blind, controlled randomized clinical study on 78 newly diagnosed but untreated mild to moderate hypertensive subjects conducted by Nwachukwu et al. (2015) observed a significant decreased in ACE activity compared with the placebo after the ingestion for four weeks of 150 mg/kg/day of an aqueous extract of Hibiscus sabdariffa rich in anthocyanins [47].

Homocysteine levels remained unchanged after the intervention with the different food products. To the best of our knowledge, very few clinical trials have examined the effects of flavanols and anthocyanins on homocysteine levels, without showing significant changes. A noteworthy study is that carried out by Grassi et al. (2008) on 19 subjects with hypertension and impaired glucose tolerance [48]. In this randomized, cross-over clinical trial, the consumption of 100 g/d of flavanol-rich dark chocolate (1008 mg/d of total phenols) for 15 days decreased insulin resistance, SBP, DBP and serum levels of TC and LDL-C, and increased insulin sensitivity, β-cell function and FMD levels compared with a flavanol-free white chocolate. However, the levels of homocysteine remained unchanged after the intervention with the two chocolates [49], in agreement with the results obtained in our work using a lower dose of flavanols over a longer intervention period and in a healthy population. In relation to anthocyanins and their effects on homocysteine levels, a randomized clinical trial carried out by Duthie et al. (2006) on 20 healthy female volunteers, the supplementation with 750 mL/day of cranberry juice (850 mg/day of total phenols) failed to significantly reduce serum levels of this metabolite after two weeks [49]. These data suggest that neither anthocyanins nor flavanols affected homocysteine metabolism and that the doses implemented were not enough to reach the concentrations necessary to produce a significant effect.

In addition, NO showed no significant changes with any of the different food products, with only a slight increase in the C and RB+C groups after the intervention. There is some scientific evidence in humans that supports the increase of NO levels in serum after acute consumption of cocoa flavanols in healthy subjects [50,51,52]. However, there is some conflicting data in this regard, as the study of Persson et al. (2011) did not observe significant changes in NO concentrations in serum after the consumption of 75 g of dark chocolate [44]. Long-term studies have also shown no significant changes in NO concentrations after the consumption of flavanol-rich foods [52,53]. In a recent randomized clinical trial conducted by Hollands et al. (2018), 42 healthy subjects were given a single dose of an apple flavanol extract (70 mg monomeric flavanols), a double dose of this extract (140 mg monomeric flavanols), an apple procyanidins extract (6.5 mg monomeric flavanols), or placebo capsules once daily for four weeks, after which the concentrations of NO did not exhibit significant changes compared with the placebo [53]. In the case of anthocyanins, we found more contradictions, as in vivo studies with rats have shown attenuate endothelial dysfunction through an increase in NO synthesis [54]. However, clinical trials with anthocyanin-rich foods have failed to show an increase in NO concentrations in blood, especially in subjects with an increased risk of CVD. For example, in a randomized, open label, two-arm, cross-over clinical trial carried out by Hollands et al. (2018) on 41 overweight subjects, the administration of 500 mL/day of blood orange juice, providing 50 mg of anthocyanins per day, for 28 days did not show an improvement in cardiovascular biomarkers, including NO, TC, HDL-C, LDL-C, TG, glucose, CRP, aortic SBP and DBP or carotid-femoral and brachial-ankle pulse compared with a standard orange juice without anthocyanins [55]. Another noteworthy study is the randomized, double-blind and parallel clinical trial conducted by Curtis et al. (2019) on 115 subjects with metabolic syndrome who consumed 150 g or 75 g of fresh blueberries rich in anthocyanins (364 mg and 182 mg of anthocyanin, respectively), or a placebo for six months. After the intervention, they observed an improvement in endothelial function, FMD, systemic arterial stiffness and an increase in cyclic guanosine monophosphate with the ingestion of 150 g fresh blueberries, compared with the placebo. However, insulin resistance, pulse wave velocity, blood pressure and NO remained unchanged after the three interventions [56]. Knowing that other flavonoids, such as hesperidin, have exhibited some stimulating effects on NO synthesis in vitro with an improvement in endothelial function [41], this suggested that both anthocyanins and flavanols could improve endothelial function by increasing underlying NO bioactivity and maintaining healthy concentrations of NO in blood. This theory is reinforced by the conclusions reached in the work of Curtis et al., as increasing circulating levels of cyclic guanosine monophosphate reflect the activity of soluble guanylate cyclase in vascular smooth muscle, which is stimulated by endothelial NO [56].

TMAO is a well-known gut microbiota-dependent metabolite with pro-atherogenic effects, enhancing the development of atherosclerosis in animal models and is associated with increased CVD risk in humans [26]. As a product of the gastrointestinal tract metabolism of dietary nutrients, such as choline, changes in the dietary pattern could influence TMAO production, and thus reduce blood vessel damage and CVD risk. Scientific evidence in this subject also shows that some bioactive compounds present in diet, such as dietary fiber and resveratrol, reduce TMAO levels and atherosclerotic plaque formation induced by TMAO [57]. In our work, we observed a significant reduction in TMAO levels in blood after the 12-week intervention with 2.5 g/day of cocoa product, which constitutes an intake of 9.59 mg/day of flavanols. In vivo studies with mice have demonstrated that food sources of catechin, such as oolong tea and citrus peel, reduce hepatic FMO3 expression, and thus reduce TMAO levels [58]. As far as we know, this is the first study that demonstrates that long-term intake of flavanol-rich cocoa significantly reduces TMAO levels in humans. In a short-term crossover clinical trial carried out by Angieletta et al. (2018) on 20 obese adults, the supplementation with 28 g of cocoa powder (30 mg/day of flavanols) and 1.2 g of green tea for five days did not cause significant changes in TMAO levels and TMA precursors (choline, carnitine and betaine) [59]. This difference in results could be due to the difference in intervention time (12 weeks vs. 5 days). In this sense, our hypothesis was that cocoa flavanols reduce TMAO production through gut microbiota modulation, and therefore five days could be too short to produce significant changes in gut microbiota metabolism. This decrease in TMAO levels after the intervention with cocoa powder seems reasonable if we consider that it was the treatment exhibiting the largest increase in polyphenol levels corrected by creatinine at the end of the intervention. Additionally, we found a significant negative correlation in women between TMAO blood levels and polyphenol levels corrected by creatinine, and such higher polyphenol levels were associated with lower levels of TMAO at the end of the intervention. The lack of statistical signification in men could be due to the difference between the number of male and female volunteers (17 vs. 42, respectively), but also to sex-related differences in polyphenol metabolism and distribution, as a study in rats found that female rats showed twice the amount of flavanol metabolites in plasma than male rats [60].

Along with the study of metabolites related to cardiovascular health, we also performed an analysis of vascular function by FMD and blood pressure values. FMD values at the start of the intervention were between 8 and 9% and are considered healthy for this population group, as values over 6.5% protect against CVD [61]. After the intervention, we observed an increase in FMD values only in the C group. This increase could be associated with the significant reduction in TMAO and the increase in polyphenol levels. However, we did not find a correlation between FMD values and these two parameters at the end of the intervention. This increase in FMD values occurred without significant changes in NO concentration and ACE activity, although we could observe a slight increase in NO with coco powder. These results are consistent with the current scientific evidence, as it has been observed that flavanol intake can prevent endothelium dysfunction and help maintain healthy blood pressure levels [16,17,18,48,62]. For example, when hypertensive volunteers consumed 100 g/d of flavanol-rich dark chocolate (1008 mg/d of total phenols) for 15 days, FMD values and insulin sensitivity increased, and SBP, DBP, TC and LDL-C levels decreased compared with a flavanol-free white chocolate [48]. Similar results were obtained in healthy volunteers, in a randomized, controlled, double-masked, parallel-group clinical trial conducted by Heiss et al. (2015) on 22 young (<35 years) and 20 elderly (50–80 years) males. The intake of a high flavanol cocoa extract (450 mg of flavanols) twice a day for two weeks significantly improved FMD values and decreased DBP in both population groups and decreased SBP only in the elderly volunteers [62]. Anthocyanins have also been proven to be effective against endothelium dysfunction [20,21,24,55,56,63]. However, the red berry mixture and the combination of both produced no changes in FMD values. As stated above, our results could be influenced by the low dose of flavanols and anthocyanins that might not be high enough to reach clinical significance. Consumption of 364 mg of anthocyanin from fresh blueberries has been proven to statistically increase FMD, HDL-C and Apolipoprotein A1 values in patients with metabolic syndrome, but not SBP and DBP [56]. In healthy subjects, a randomized, double-blind, placebo-controlled clinical trial conducted by Novotny et al. (2015) demonstrated that the consumption of 240 mL of a low-calorie cranberry juice (173 mg total phenols) twice a day for eight weeks significantly reduced DBP, TG, CRP and fasting plasma glucose, but not SBP [63].

SBP, DBP, TC, TG, HDL-C, TC/HDL, glucose, CRP and iron remained unchanged with all the food products and around the values established as healthy by the medical community. Remarkably, the creatinine and uric acid concentrations significantly decreased in the C group after the intervention. These results are coherent with previous studies with Camellia sinensis flavanols, which have a proven uric acid lowering effect through the modulation of both xanthine oxidase and urate excretion [64]. Unexpectedly, we observed an increase in HR at the end of the intervention in the RB and C groups. Methylxanthines, such as theobromine present in chocolate, have been described in the literature to increase HR in healthy subjects [65]. However, we did not find any study in the literature that had observed an increase in HR after a supplementation with anthocyanins. Given that HR can be easily altered by external factors, that the values after the intervention remained in the normal, healthy range and that no other cardiovascular parameters showed any significant changes, we did not consider this increase clinically relevant.

Both SBAs and SCFAs are products of the microbiota metabolism [25], and therefore changes in its composition and function may produce changes in the concentrations of these biliary acids. Flavanols [29] and anthocyanins [30] have been shown to modify gut microbiota composition and possibly to be associated with changes in SCFA and SBA concentrations in blood. In our trial, DCA concentrations significantly increased after the 12-week intervention with the red berry product. DCA is a bile acid derived from cholic acid by microbiota transformation and studies suggest that it is associated with numerous detrimental effects, such as inflammation, immune dysregulation, dyslipidemia, decreased insulin sensitivity and vascular calcification. A recent prospective observational cohort study on 3147 subjects with chronic renal insufficiency found that high levels of DCA in serum were associated with a higher risk of end-stage kidney disease and all-cause mortality but not with atherosclerosis and heart failure [66]. We also observed a positive correlation between DCA and glucose and creatinine concentrations, thus indicating that DCA could affect the body metabolism in a negative way. In fact, most of the correlations found between bile acids and cardiovascular parameters show a negative effect of increasing concentrations of these metabolites on cardiovascular health, as previously described [32]. To our knowledge, this is the first study on humans that has tried to find an association between the regular intake of food products rich in flavanols and/or anthocyanins and changes in BA and SCFA serum concentrations. However, in vivo studies with mice observed that the intake of flavonoid-rich foods, such as cranberries [67] and Pu-erh tea [68], promoted fecal excretion of cholic acid and DCA and reduced the serum amount of these two BAs by targeting the regulation of intestinal microorganisms. The lack of results in our work could be due to external factors, such as dietary habits, age, as DCA tend to be increased in older individuals [67], or sex, as it has been shown that, in mice, aging increases hepatic and colonic DCA, especially in males [69]. In the case of the SCFAs, we observed that at the end of the intervention, all metabolites, except BUT in the C and RB+G groups, tended to increase. However, we only found a significant increase in the FIA after the intervention with the red berry anthocyanins and the cocoa flavanols. This indicates an increase in carbohydrate fermentation, and therefore a higher synthesis of SCFAs by the gut microbiota, thus explaining the observed increase in SCFAs with the two food products. SCFAs are a crucial compound in the interaction between the gut microbiota and host metabolism by regulating a vast variety of biological processes, such as adipogenesis, energy metabolism, appetite control, intestinal cellular homeostasis, gut motility, glucose metabolism, inflammation, and central and sympathetic nervous system function. One way in which SCFAs could improve cardiovascular health is by protecting against obesity by increasing energy expenditure, anorexic hormone production and improving appetite regulation [70]. Studies that examine the direct actions of SCFAs on the cardiovascular system are scarce, but a recent study found that a high abundance of butyrate-producing bacteria, such as Lachnospiraceae, Ruminococcaceae and Acidaminococcaceae families, is associated with lower blood pressure in pregnant women with obesity [71]. In fact, we found a negative correlation in women between the FIA value and DBP and TC/HDL ratio, thus indicating that high levels of carbohydrate fermentation are associated with lower blood pressure levels and a better lipid profile, reinforcing the statement that SCFAs protect against CVD.

One of the main objectives of the present trial was to evaluate the efficiency of a mixture of red berry anthocyanins and cocoa flavanols in enhancing the positive effects of these two polyphenol groups on the cardiovascular system. In a previous paper by our team [7], we demonstrated that the regular intake of red berries, cocoa and a mixture of both improved executive functions. We detected a reduction in the time needed to start and finish the Tower of London test in all groups, but the decrease in time to finish the neurocognitive task was more pronounced in the intervention with the RB+C group. Furthermore, the RB+C group showed an improvement in the Verbal Learning Test Spain-Complutense (TAVEC) and in perceptual speed, accuracy and speed in processing punctuation in total. These improvements were independent of changes in the concentrations of the brain-derived neurotrophic factor (BDNF) and nerve growth factor receptor (NGF-R). Considering the results obtained in the present work, the improvement in executive function could be explained by the enhancement in cardiovascular function after a regular intake of red berry anthocyanins and cocoa flavanols, as it has been previously described that one of the ways that polyphenols maintain a healthy cognitive function is by increasing cerebral blood flow [72]. However, neither of the cardiovascular biomarkers studied showed a statistically significant change after the regular intake of the mixture food product. An explanation for this lack of effect may be an unknown antagonistic interaction between red berry anthocyanins and cocoa flavanols, as it has been observed in vitro that dimeric structures containing both anthocyanins and flavanols can cross the intestinal barrier but with lower efficiency than the isolated compounds [73]. Furthermore, it is important to take into account that SCFAs have their own neuroprotective properties and could prevent cognitive impairment, as they can cross the blood–brain barrier via monocarboxylate transporters and improve neuronal homeostasis and function. They have been shown to act at this level by promoting neurogenesis, increasing the concentrations of neurotrophic factors and serotonin synthesis, reducing neuroinflammation by affecting glial cell morphology and function and maintaining the integrity of the blood–brain barrier by increasing expression of tight junction proteins [74].

Therefore, our study showed that both red berry anthocyanins and cocoa flavanols could modulate the gut microbiota metabolism in a positive way, and as a result have a protective effect on cardiovascular health in healthy adults. The intervention with cocoa flavanols showed better results, significantly lowering the levels of TMAO and uric acid and increasing FMD values. Additionally, we found a negative correlation between TMAO concentrations and polyphenol levels in blood, showing that individuals in whom higher concentrations of polyphenols were found after the intervention presented lower levels of TMAO. However, it should be noted that our study had some important limitations, including that the number of subjects was slightly low considering that it was a parallel study and that there was no control group included. This lack of control group was due to the difficulty in finding a food product that could serve as a placebo for cocoa and red berries. It is also important to mention that we did not perform a direct analysis of the microbiome but an analysis of the metabolites derived from the gut microbiota as markers of possible changes in its composition. Future works might consider expanding the intervention time in order to clarify the effects of both compounds on SCFAs, SBAs and other cardiovascular parameters, such as ACE activity and NO concentrations. Moreover, an in-depth study of gut microbiota regulation is needed. In this sense, further studies should be carried out to better understand how the regular intake of anthocyanins and flavanols modulate the gut microbiota composition.

## 5. Conclusions

Regular intake of cocoa flavanols improves cardiovascular health by reducing TMAO and uric acid levels and increasing FMD values, additionally showing a direct association with polyphenol levels in serum. Concentrations of TMAO negatively correlated with polyphenol levels at the end of the intervention. Both cocoa flavanols and red berry anthocyanins improve gut microbiota metabolism by increasing carbohydrate fermentation, and therefore increase the synthesis of SCFAs. This increase in microbiota fermentation correlated negatively with SBP, DBP and TC/HDL ratio. A slight increase in SCFAs was observed with the three products at the end of the intervention but without arriving at statistical significance. Overall, increasing polyphenol rich foods in the diet might constitute a good strategy to prevent or postpone the cardiovascular morbidity associated with ageing. Further placebo controlled studies are needed to better understand the mechanisms implied in the potential cardiovascular protective and prebiotic effects of cocoa flavanols and red berry anthocyanins.

## Figures and Tables

**Table 1:** Total protein, ACE, NO, homocysteine and TMAO levels in serum in each group and by visit.

	RB			C			RB+C			*p*-Value **
	Baseline	12 w	*p*-Value *	Baseline	12 w	*p*-Value *	Baseline	12 w	*p*-Value *	
Total protein (mg/mL)	84.88 ± 31.13	86.59 ± 36.06	0.52	94.69 ± 30.13	95.63 ± 31.98	0.97	87.51 ± 29.5	84.61 ± 35.57	0.30	0.49
ACE/total protein	0.74 ± 0.52	0.78 ± 0.39	0.69	0.90 ± 0.48	0.82 ± 0.37	0.43	0.80 ± 0.56	0.87 ± 0.47	0.23	0.91
NO (µM)	27.91 ± 17.58	27.04 ± 13.86	0.50	22.42 ± 12.26	26.21 ± 17.54	0.28	32.57 ± 17.26	36.44 ± 24.16	0.29	0.40
Homocysteine (mg/dL)	15.20 ± 3.15	15.16 ± 4.11	0.79	14.11 ± 3.68	14.49 ± 4.06	0.79	15.91 ± 3.42	15.62 ± 3.58	0.66	0.46
TMAO (µM)	9.71 ± 2.26	9.34 ± 2.45	0.52	10.10 ± 3.09	8.72 ± 2.58	0.03	9.97 ± 2.29	10.34 ± 1.91	0.41	0.14
FMD (%)	7.9 ± 5.12	9.14 ± 5.10 ^b^	0.95	9.47 ± 3.2	14.78 ± 0.89 ^a^	0.03	8.89 ± 4.14	10.41 ± 3.75 ^a,b^	0.24	0.04

* Significance within groups, ** significance between groups end of the intervention; ^a^, ^b^, significant differences between groups; data expressed as mean ± standard deviation (SD); significant difference at *p*-value < 0.05. Angiotensin-converting enzyme (ACE), Nitric oxide (NO), trimethylamine N-oxide (TMAO), Flow-mediated dilation (FMD).

**Table 2:** Glucose, iron, C-reactive protein and lipid metabolism biomarkers in serum in each group and by visit.

	RB			C			RB+C			*p*-Value **
	Baseline	12 w	*p*-Value *	Baseline	12 w	*p*-Value *	Baseline	12 w	*p*-Value *	
Glucose (mg/dL)	88.89 ± 14.17	87.89 ± 15.50	0.94	80.79 ± 11.46	80.37 ± 13.1	0.89	83.5 ± 12.39	81.11 ± 13.65	0.34	0.22
Iron (mg/dL)	95.05 ± 28.10	89.55 ± 22.52	0.54	102.37 ± 27.10	100.53 ± 29.46	0.64	91.94 ± 33.74	90.28 ± 33.29	0.78	0.20
TC (mg/dL)	198.88 ± 36.72	208.12 ± 28.17	0.07	197.65 ± 23.23	202.55 ± 32.10	0.27	207.88 ± 28.07	209.94 ± 28.81	0.92	0.76
TG (mg/dL)	103.5 ± 51.48	107.5 ± 48.20	0.82	109.44 ± 42.55	103.22 ± 38.87	0.46	108.18 ± 52.14	121.0.6 ± 49.69	0.38	0.56
HDL-C (mg/dL)	62.45 ± 15.85	65.0 ± 12.65	0.21	59.50 ± 14.60	60.60 ± 16.41	0.28	62.61 ± 14.08	65.22 ± 14.91	0.72	0.67
TC/ HDL	3.46 ± 0.95	3.47 ± 0.84	0.86	3.53 ± 0.94	3.51 ± 0.79	0.71	3.54 ± 0.88	3.47 ± 0.75	0.41	0.96
CRP (mg/mL)	0.16 ± 0.20	0.17 ± 0.22	0.75	0.22 ± 0.25	0.15 ± 0.15	0.30	0.11 ± 0.13	0.15 ± 0.14	0.75	0.80

* Significance within groups, ** significance between groups at the end of the intervention; data expressed as mean ± standard deviation (SD); significant difference at *p*-value < 0.05. Total cholesterol (TC), triglycerides (TG), C-reactive protein (CRP).

**Table 3:** Creatinine and uric acids levels in serum in each group and by visit.

	RB			C			RB+C			*p*-Value **
	Baseline	12 w	*p*-Value *	Baseline	12 w	*p*-Value *	Baseline	12 w	*p*-Value *	
Creatinine (mg/dL)	0.68 ± 0.12	0.71 ± 0.13	0.17	0.74 ± 0.15	0.7 ± 0.13	0.03	0.7 ± 0.13	0.69 ± 0.12	0.53	0.91
Uric acid (mg/dL)	4.72 ± 1.01	4.7 ± 1.04	0.43	4.91 ± 0.82	4.53 ± 0.77	0.01	4.43 ± 1.16	4.14 ± 0.94	0.05	0.08

* Significance within groups, ** significance between groups at the end of the intervention; data expressed as mean ± standard deviation (SD); significant difference at *p*-value < 0.05.

**Table 4:** Polyphenol levels corrected by creatinine in each group and by visit.

	RB			C			RB+C			*p*-Value **
	Baseline	12 w	*p*-Value *	Baseline	12 w	*p*-Value *	Baseline	12 w	*p*-Value *	
polyphenol/ creatinine (mg/mL)	120.16 ± 55.53	151.9 ± 62.30 ^a,b^	0.06	158.78 ± 60.49	199.7 ± 86.65 ^a^	0.03	119.57 ± 40.65	130.96 ± 64.74 ^b^	0.08	0.04

* Significance within groups, ** significance between groups at the end of the intervention; ^a^, ^b^, significant differences between groups; data expressed as mean ± standard deviation (SD); significant difference at *p*-value < 0.05.

**Table 5:** Bile acids and sum of primary, secondary and total bile acids concentrations in ng/mL by each group and by visit.

	RB			C			RB+C			*p*-Value **
	Baseline	12 w	*p*-Value *	Baseline	12 w	*p*-Value *	Baseline	12 w	*p*-Value *	
CDCA	83.11 ± 68.06	220.81± 219.66 ^a^	0.08	97.58 ± 85.64	66.41 ± 82.74 ^b^	0.19	79.62 ± 124.37	108.24 ± 91.76 ^a,b^	0.17	0.03
DCA	116.74 ± 79.98	249.98 ± 193.48	0.03	172.88 ± 95.94	121.47 ± 75.16	0.16	125.86 ± 120.74	157.54 ± 124.72	0.40	0.11
GDCA	184.78 ± 178.87	260.22 ± 231.51	0.19	230.21 ± 186.72	153.71 ± 156.95	0.17	162.77 ± 135.01	171.93 ± 106.43	0.63	0.15
GCA	60.09 ± 37.95	71.05 ± 56.67	0.94	57.05 ± 40.78	61.16 ± 57.69	0.89	66.84 ± 63.81	87.97 ± 58.74	0.28	0.26
TDCA	28.89 ± 25.41	29.12 ± 19.51	0.74	25.69 ± 16.1	25.82 ± 24.64	0.65	17.02 ± 9.59	16.73 ± 10.78	1.00	0.27
TCA	16.29 ± 12.8	19.73 ± 14.05	0.55	13.64 ± 7.22	15.15 ± 7.12	0.72	10.09 ± 2.91	13.90 ± 6.72	0.22	0.74
GCDCA	295.89 ± 197.57	489.93 ± 388.54	0.06	410.84 ± 241.78	328.83 ± 243.8	0.42	347.41 ± 277.51	327.60 ± 227.09	0.89	0.20
GUDCA	26.04 ± 15.27	39.40 ± 33.44	0.29	45.46 ± 36.73	29.76 ± 19.87	0.29	32.63 ± 27.15	29.04 ± 17.48	0.67	0.74
TCDCA	52.50 ± 40.96	72.13 ± 69.25	0.65	40.69 ± 31.65	54.75 ± 44.07	0.54	28.40 ± 16.55	29.93 ± 17.84	0.82	0.16
GLCA	37.28 ± 39.4	39.34 ± 44.52	0.71	26.93 ± 16.55	24.31 ± 21.42	0.35	33.07 ± 28.68	24.54 ± 17.13	1.00	0.34
Total BA	979.13 ± 463.5	979.67 ± 472.94	0.74	1313.95 ± 466.84	1079.89 ± 505.95	0.38	953.84 ± 650.29	1045.18 ± 476.36	0.46	0.83
Primary	521.27 ± 242.13	791.44 ± 602.53	0.32	655.36 ± 297.03	591.30 ± 318.21	0.85	512.03 ± 362.07	618.76 ± 295.03	0.21	0.87
Secondary	400.66 ± 254.56	411.46 ± 230.52	0.68	569.47 ± 209.81	430.77 ± 241.53	0.36	422.52 ± 278.55	418.03 ± 215.11	0.82	0.98

* Significance within groups, ** significance between groups at the end of the intervention, ^a^, ^b^, significant differences between groups; data expressed as mean ± standard deviation (SD); significant difference at *p*-value < 0.05. Chenodeoxycholic acid (CDCA), Deoxycholic acid (DCA), Glyco-deoxycholic acid (GDCA), Glycolic acid (GCA), Tauro-deoxycholic acid (TDCA), Taurocholic acid (TCA), Glycokeno-deoxycholic acid (GCDCA), Glycourso-deoxycholic acid (GUDCA), Tauroquene-deoxycholic acid (TCDCA), Glyco-lithocholic acid (GLCA).

**Table 6:** Short chain fatty acids concentrations, sum and fermentation index in ng/mL by each group and by visit.

	RB			C			RB+C			*p*-Value **
	Baseline	12 w	*p*-Value *	Baseline	12 w	*p*-Value *	Baseline	12 w	*p*-Value *	
ACE	0.40 ± 0.13	0.52 ± 0.1	0.06	0.43 ± 0.12	0.52 ± 0.19	0.12	0.41 ± 0.17	0.53 ± 0.24	0.14	0.78
PRO	0.02 ± 0.01	0.03 ± 0.01	0.28	0.03 ± 0.01	0.03 ± 0.01	0.60	0.03 ± 0.01	0.03 ± 0.01	0.27	0.38
ISOB	0.01 ± 0.00	0.01 ± 0.00	0.09	0.01 ± 0.00	0.01 ± 0.00	0.45	0.01 ± 0.00	0.01 ± 0.00	0.24	0.85
BUT	0.03 ± 0.01	0.03 ± 0.01	0.71	0.03 ± 0.01	0.03 ± 0.01	0.06	0.03 ± 0.01	0.03 ± 0.01	0.97	0.39
ISOV	0.01 ± 0.00	0.01 ± 0.00 ^a,b^	0.42	0.01 ± 0.00	0.01 ± 0.00 ^a^	0.41	0.01 ± 0.00	0.01 ± 0.00 ^b^	0.34	0.00
VAL	0.002 ± 0.008	0.001 ± 0.001	0.21	0.002 ± 0.007	0.002 ± 0.001	0.48	0.002 ± 0.001	0.002 ± 0.001	1.00	0.53
CAP	0.01 ± 0.00	0.01 ± 0.00 ^a^	0.13	0.01 ± 0.00	0.01 ± 0.00 ^a,b^	0.62	0.01 ± 0.00	0.01 ± 0.00 ^b^	0.44	0.03
HEP	0.01 ± 0.00	0.00 ± 0.00	0.32	0.01 ± 0.00	0.01 ± 0.00	0.37	0.01 ± 0.00	0.00 ± 0.00	0.66	0.48
Total SCFA	0.48 ± 0.15	0.61 ± 0.11	0.06	0.52 ± 0.13	0.60 ± 0.21	0.16	0.48 ± 0.18	0.60 ± 0.25	0.22	0.79
FIA	0.01 ± 0.00	0.01 ± 0.00	0.04	0.01 ± 0.00	0.01 ± 0.00	0.04	0.01 ± 0.00	0.01 ± 0.00	0.33	0.38
FIB	0.01 ± 0.00	0.01 ± 0.00	0.46	0.02 ± 0.00	0.02 ± 0.00	0.86	0.01 ± 0.00	0.01 ± 0.00	1.00	0.85

* Significance within groups, ** significance between groups at the end of the intervention, ^a^, ^b^ significant differences between groups, data expressed as mean ± standard deviation (SD); significant difference at *p*-value < 0.05. Acetic (ACE), propionic (PRO), iso-butyric (ISOB), butyric (BUT), isovaleric (ISOV), valeric (VAL), caproic (CAP), heptanoic (HEP), fermentation index A (FIA), fermentation index B (FIAB).

**Table 7:** Sex-segmented bivariate correlations between biochemical parameters and polyphenol levels corrected by creatinine.

	Polyphenols Levels			
	Men		Women	
	R	*p*-Value	R	*p*-Value
ACE/total protein	0.34	0.27	0.12	0.50
NO (µM)	−0.36	0.19	−0.17	0.32
Homocysteine (mg/dL)	−0.04	0.88	−0.11	0.52
TMAO (µM)	−0.36	0.23	−0.57	0.00
Systolic pressure (mmHg)	0.19	0.51	0.18	0.29
Diastolic pressure (mmHg)	0.37	0.17	0.26	0.11
Heart rate (bpm)	0.32	0.25	−0.16	0.93
FMD (%)	0.14	0.75	−0.12	0.69
Glucose (mg/dL)	−0.06	0.85	0.10	0.56
Iron (mg/dL)	−0.09	0.75	0.2	0.24
TC (mg/dL)	−0.22	0.45	0.19	0.29
TG (mg/dL)	−0.15	0.61	−0.25	0.15
HDL-C (mg/dL)	−0.21	0.46	0.29	0.07
TC/ HDL	−0.00	0.99	−0.16	0.35
CRP (mg/mL)	0.35	0.22	−0.01	0.95
Creatinine (mg/dL)	0.14	0.62	−0.28	0.09
Uric acid (mg/dL)	0.1	0.73	0.07	0.68

Significant difference at *p*-value < 0.05. Angiotensin-converting enzyme (ACE), Nitric oxide (NO), trimethylamine N-oxide (TMAO), flow-mediated dilation (FMD), Total cholesterol (TC), triglycerides (TG), C-reactive protein (CPR).

**Table 8:** Sex-segmented bivariate correlations between short chain fatty acids concentrations in ng/mL and polyphenol levels corrected by creatinine.

	Polyphenols Levels			
	Men		Women	
	R	*p*-Value	R	*p*-Value
ACE	0.36	0.25	0.19	0.93
PRO	−0.04	0.90	−0.05	0.79
ISOB	−0.09	0.77	−0.16	0.44
BUT	0.55	0.05	−0.20	0.31
ISOV	−0.10	0.75	0.08	0.68
VAL	0.178	0.56	0.09	0.65
CAP	0.36	0.23	0.18	0.37
HEP	0.11	0.73	0.08	0.68
Total SCFA	0.38	0.22	0.00	0.99
FIA	0.01	0.98	0.07	0.72
FIB	−0.16	0.63	−0.07	0.74

Significant difference at *p*-value < 0.05. Acetic (ACE), propionic (PRO), iso-butyric (ISOB), butyric (BUT), isovaleric (ISOV), valeric (VAL), caproic (CAP), heptanoic (HEP), fermentation index A (FIA), fermentation index B (FIB).

**Table 9:** Sex-segmented bivariate correlations between bile acids concentrations in ng/mL and polyphenol levels corrected by creatinine.

	Polyphenols Levels			
	Men		Women	
	R	*p*-Value	R	*p*-Value
CDCA	0.062	0.85	−0.289	0.12
DCA	0.215	0.48	−0.161	0.38
GDCA	−0.119	0.71	−0.281	0.11
GCA	−0.179	0.58	−0.319	0.08
TDCA	0.541	0.17	0.057	0.77
TCA	−0.054	0.88	0.047	0.82
GCDCA	−0.197	0.54	−0.056	0.76
GUDCA	−0.356	0.26	−0.228	0.24
TCDCA	−0.166	0.61	0.19	0.32
GLCA	−0.267	0.40	−0.7	0.72
Total BA	0.126	0.79	−0.068	0.81
Primary	−0.214	0.58	−0.153	0.52
Secondary	0.39	0.34	−0.267	0.27

Significant difference at *p*-value < 0.05. Chenodeoxycholic acid (CDCA), Deoxycholic acid (DCA), Glyco-deoxycholic acid (GDCA), Glycolic acid (GCA), Tauro-deoxycholic acid (TDCA), Taurocholic acid (TCA), Glycokeno-deoxycholic acid (GCDCA), Glycourso-deoxycholic acid (GUDCA), Tauroquene-deoxycholic acid (TCDCA), Glyco-lithocholic acid (GLCA).

## Data Availability

Data is contained within the article and Supplementary Materials. The data presented in this study are available on request from the corresponding author. The data are not publicly available due to privacy issues.

## Supplementary Materials

The following supporting information can be downloaded at: https://www.mdpi.com/article/10.3390/nu15102299/s1, Table S1: Nutritional composition of the red berries mixture, Table S2: Nutritional composition of cacao powder, Table S3: Demographics and characteristics of the participants at enrollment in each group, Table S4: Blood pressure and heart rate values in each group and by visit, Table S5: *p*-value from the comparison between diets at the baseline.
