# Supplementary material for: Regular Consumption of Cocoa and Red Berries as a Strategy to Improve Cardiovascular Biomarkers via Modulation of Microbiota Metabolism in Healthy Aging Adults
Source: Nutrients. 2023 May 13;15(10):2299. doi: 10.3390/nu15102299 (PMC10223313; doi:10.3390/nu15102299)
Supplement: Supplementary file 1 [file nutrients-15-02299-s001.zip › nutrients-2319312-supplementary.pdf]

Table S1. Nutritional composition of the red berries mixture.

	Per 100g	%ARI	Per doses (5g)	%ARI per 5g
Energy value (kcal)	355.5	17.8	17.8	0.9
Fat (g)	2.7	3.8	0.1	0.2
Saturated (g)	0.1	0.6	0.0	0.0
Carbohydrates (g)	86.5	33.3	4.3	1.7
Sugars (g)	35.4	39.3	1.8	2.0
Fibre (g)	39.9	-	2.0	-
Protein (g)	7.3	14.6	0.4	0.7
Salt (g)	0.2	0.3	0.01	0.0

Data adapted from the one provided by Salengei® (Barcelona, Spain). ARI: Adult Reference Intake (8400 KJ/ 2000 Kcal).

Table S2. Nutritional composition of cacao powder.

	Per 100g	%ARI per 100g	Per doses (2.5g)	%ARI per 2.5g
Energy value (kcal)	353	18	8.8	0.5
Fat (g)	11	16	0.3	0.4
Saturated (g)	6.8	34	0.2	0.9
HC (g)	20	8	0.5	0.2
Sugars (g)	2.45	3	0.1	0.1
Fibre (g)	32	-	0.8	-
Protein (g)	27.8	56	0.7	1.4
Salt (g)	0.1	2	0.003	0.1
Potassium (mg)	1524	76	38.1	1.9
Calcium (mg)	128	16	3.2	0.4
Phosphorus (mg)	734	105	18.4	2.6
Magnesium (mg)	499	133	12.5	3.3
Iron (mg)	13.9	99	0.3	2.5
Zinc (mg)	6.8	68	0.2	1.7
Copper (mg)	4	400	0.1	10
Manganese (mg)	38	190	1	4.8
Selenium (µg)	14.3	26	0.4	0.7

Data adapted from the one provided by Salengei® (Barcelona, Spain). ARI: Adult Reference Intake (8400 KJ/ 2000 Kcal).

Table S3. Demographics and characteristics of the participants at enrollment in each group.

	Total	RB	C	RB+C	p-value
N	59	20	20	19	
Females	71.2%	60%	85%	68.4%	0.22
Age (years)	57.8 ± 6.94	56.4 ± 4.14	59.15 ± 9.08	57.84 ± 6.76	0.46
Height (m)	1.65 ± 0.08	1.67 ± 0.10	1.62 ± 0.08	1.66 ± 0.06	0.14
BMI (kg/m^2)	25.79 ± 3.52	25.24 ± 2.62	26.23 ± 4.44	25.91 ± 3.38	0.67
Smokers (%)	20%	15%	20%	26.3%	0.80
Medication (%)	42.37%	50%	40%	36.84%	0.72

Data expressed as mean ± standard deviation (SD); significant difference at p-value < 0.05.

Table S4. Blood pressure and heart rate values in each group and by visit.

	RB			C			RB+C			p-value**
	Baseline	12 w	p-value*	Baseline	12 w	p-value*	Baseline	12 w	p-value*	
Systolic pressure (mmHg)	123.1 ± 16.30	124.9 ± 16.30	0.34	126.2 ± 20.80	122.2 ± 18.50	0.22	118.4 ± 16.30	118.8 ± 16.10	0.89	0.80
Diastolic pressure (mmHg)	83 ± 11.10	83.4 ± 13.50	0.71	83.6 ± 12.80	81.2 ± 8.40	0.18	78.4 ± 10.80	80 ± 10.4	0.43	0.40
Heart rate (bpm)	64.3 ± 9.79	69.97 ± 12.28	0.00	67.77 ± 10.71	73.06 ± 12.21	0.01	65.08 ± 7.88	66.99 ± 9.51	0.34	0.18

* Significance within groups, ** significance between groups at the end of the intervention, a, b, c significant differences between groups data expressed as mean ± standard deviation (SD); significant difference at p-value < 0.05.

Table S5. P-value from the comparison between diets at the baseline.

Biomarkers of cardiovascular health	
Compound	p-value
TMAO (μM)	0.827
Total Protein (mg/ml)	0.718
ACE/total protein	0.344
NO (μM)	0.294
FMD (%)	0.871
Polyphenol/ creatinine (mg/ml)	0.451
Systolic pressure	0.671
Diastolic pressure	0.220
Heart rate	0.278
Homocysteine	0.189
Glucose	0.326
Creatinine	0.445
Uric acid	0.280
iron	0.581
Cholesterol	0.317
TG	0.722
HDL-C	0.800
TC/HDL	0.898
PCR	0.698

Short chain fatty acids

Compound	p-value
ACE	0.701
PROV	0.491
ISOB	0.090
BUT	0.315
ISOV	0.574
VAL	0.981
CAP	0.650
HEP	0.179
TOTAL	0.584
FIA	0.730
FIB	0.322

Biliary acids

Compound	p-value
CDCA	0.224
DCA	0.139
GDCA	0.548
GCA	0.904
TDCA	0.346
TCA	0.286
GCDCA	0.390
GUDCA	0.222
TCDCA	0.285
GLCA	0.931
Total BA	0.215
Primary	0.477
Secondary	0.122

Angiotensin-converting enzyme (ACE), Nitric oxide (NO), trimethylamine N-oxide (TMAO), Flow-mediated dilation (FMD), total cholesterol (TC), triglycerides (TG), C-reactive protein (CPR), acetic (ACE), propionic (PRO), isobutyric (ISOB), butyric (BUT), isovaleric (ISOV), valeric (VAL), caproic (CAP), heptanoic (HEP), fermentation index A (FIA), fermentation index B (FIAB), Chenodeoxycholic acid (CDCA), Deoxycholic acid (DCA), Glycodesoxycholic acid (GDCA), Glycolic acid (GCA), Taurodeoxycholic acid (TDCA), Taurocholic acid (TCA), Glycokenodeoxycholic acid (GCDCA), Glycoursodeoxycholic acid (GUDCA), Tauroquenedeoxycholic acid (TCDCA), Glycolithocholic acid (GLCA).
